# Effectiveness of adalimumab in the treatment of juvenile idiopathic arthritis associated with uveitis

**DOI:** 10.1186/1546-0096-12-S1-O5

**Published:** 2014-09-17

**Authors:** Elena Zholobova, Lelia Galstian, MN Nikolaeva, OY Loskutova

**Affiliations:** 1 Departament of Pediatric Rheumatology, First Moscow Medical State University I.M. Sechenov, Moscow, Russian Federation

## Introduction

Juvenile idiopathic arthritis (JIA) is a chronic autoimmune disease of children and adolescents with primary joints involvement and various manifestations. Uveitis is of extra-articular signs characterized by eye inflammation. Standard antirheumatic drugs in combination with local therapy are effective in 60%. When these drugs are ineffective, genetically engineered drugs are used in JIA with rheumatoid uveitis.

## Objectives

This prospective observation was aimed to assessing effectiveness and safety of adalimumab(ADA) in the treatment of juvenile idiopathic arthritis associated with uveitis in patients with severe JIA, prolonged disease and resistance to standard antirheumatic therapy.

## Methods

Among 27 patients with JIA and eye involvement included in the study, 13 children had oligoarticular JIA, 8 children - polyarticular, 6 patients had systemic disease. Study group included 20 girls and 7 boys. Mean age was 7.0 years, age of disease onset was 3.5 ± 2.07 years; mean disease duration before ADA administration was 5 ± 3.6. Disease onset with initial joint damage was observed in 22 children, with eye involvement - in 5 children. Prior to ADA administration most children had high (III) disease activity. Number of active joints was 10.8 ± 3.2, mean ESR was 27.0 ± 13.37 mm/h, CRP – 2.25 ± 1.1 mg/dl (ref. <0.8 mg/dl). All 27 patients had active uveitis at the moment of ADA administration. Twenty-four (89%) patients had bilateral ocular involvement, 3 patients (11%) - unilateral. Clinical Global Impression -Disease Activity (VAS) score was 76.2 ± 14.0,patient/parent score was 65.9 ± 17.1. Mean functional disability in patients before ADA administration was 2.04 ± 0.57. Prior to ADA administration all children received immunosuppressive drugs: 26 children (96%) methotrexate (MTX) 10-15mg/m^2^ of body surface per week, 14 children (52%) received SandimmuneNeoral as monotherapy or in combination with methotrexate, 10 children (37%) received oral corticosteroids (CS), 27 children (100%) received active topical treatment of uveitis.

## Results

After 6 months of adalimumab therapy, humoral activity decreasedin 24 patients (89%). ESR and CRP also substantially decreased. ACRpedi-30 was achieved in 100.0%patients, ACRpedi-50 - in 18 patients (67%), ACRpedi-70 - in 13 children (47%). Uveitis remission was achieved in 41.2% eyes; 31.4% showed a significant reduction in inflammatory activity, 27.4% had no significant dynamics. After 12 months of adalimumab therapy, number of active joints decreased from 10.8±3.2 to4.67±1.6. Mean ESR decreased from 27.0 ± 13.37 mm/h to 6±4.9 mm/h, CRP – from 2.25 ± 1.1 mg/dl to 0±0.34mg/dl, Clinical Global Impression -Disease Activity (VAS) score decreased from 76.2 ± 14 to37±12.5,patient/parent score decreased from 65.9 ± 17,1 to26 ± 15. ACRpedi-30 was achieved in 100.0% patients, ACRpedi-50 - in 20 patients (74%), ACR pedi-70 in 16 children (59.2%).

Regarding uveitis, 46,3% patients had uveitis remission, 16.7% patients–sub active process. Flares were observed in 6 patients (22,2%) often due to noncompliance of combination therapy with adalimumab and methotrexate, while if adalimumab and methotrexate was taken according to the recommended dosing regimen flares were observed only in 4 patients (14,8 %).

## Conclusion

Overall, treatment with adalimumab (Humira®) was effective in the vast majority of patients with chronic juvenile idiopathic arthritis and uveitis with high clinical and laboratory activity, and resistance to standard antirheumatic therapy.

## Disclosure of interest

None declared.

